# Identification of a persistent *Ascaris*-derived Kalirin epitope associated with chronic T cell activation in the lung

**DOI:** 10.1371/journal.pntd.0014434

**Published:** 2026-06-09

**Authors:** Yifan Wu, Leroy Versteeg, Meng-Chih Wu, Jill E. Weatherhead

**Affiliations:** 1 Department of Pediatrics, Division of Tropical Medicine, Baylor College of Medicine, Houston, Texas, United States of America; 2 Dan L. Duncan Comprehensive Cancer Center, Baylor College of Medicine, Houston, Texas, United States of America; 3 Department of Medicine, Section of Infectious Diseases, Baylor College of Medicine, Houston, Texas, United States of America; 4 William T. Shearer Center for Human Immunobiology, Baylor College of Medicine and Texas Children’s Hospital, Houston, Texas, United States of America; 5 National School of Tropical Medicine, Baylor College of Medicine, Houston, Texas, United States of America; Washington University in St Louis School of Medicine, UNITED STATES OF AMERICA

## Abstract

Ascariasis remains a dominant global health burden due to its vast prevalence and associated morbidity. The obligatory migration of *Ascaris* larvae through pulmonary tissue triggers intense type-2 inflammation which typically presents as acute allergic airway disease. Even after the parasite is eliminated, a single episode of larval migration can result in chronic lung damage and dysfunction, which may be driven by the long-term retention of helminth antigens in macrophages. However, the molecular identity of these retained antigens, and the mechanisms by which they sustain chronic T cell responses, remain unknown. In this study, we utilized immunopeptidomics to identify a retained peptide specific from *Ascaris* that is sequestered and presented by pulmonary macrophages via MHC-II. We further demonstrated that this retained peptide serves as an epitope which is associated with the development of specific T helper cell populations that persist long after the infection has cleared. These findings define a potential molecular mechanism for persistent helminth-induced immune cell activiation in the lungs and identify a retained epitope as a potential contributor to the development of chronic pulmonary inflammation following parasite elimination from the lungs.

## Author summary

Infections with the parasitic worm *Ascaris* affect hundreds of millions of people globally and are a major cause of chronic respiratory illness. During their life cycle, these parasites migrate through the host’s lungs, triggering an intense immune response that can lead to permanent tissue damage and long-term pulmonary complications. While *Ascaris* is eventually cleared from the body, the underlying inflammation often persists. Our research addresses the mechanisms by which damage continues long after the infection resolves. Using immunopeptidomics, we identified a specific molecular parasitic peptide that is retained within the lung tissue. We discovered that pulmonary macrophages present this peptide as an epitope to activate the host immune system after the infection has resolved. This persistent signal is associated with activating T cells, which may contribute to a state of chronic inflammation even in the absence of a live parasite. By identifying this retained epitope, our study provides a mechanism for how remote helminth infections contribute to chronic disease development and offers a potential target for therapeutic intervention.

## Introduction

With an estimated 500 million people affected, primarily within low- and middle-income countries, ascariasis represents the most prevalent helminthic infection worldwide. This disease imposes a staggering global burden, resulting in approximately 800,000 disability-adjusted life years lost to morbidity [1–3]. In endemic settings, infection begins in early infancy and continues throughout childhood as a series of recurrent infections [4, 5]. Transmission occurs through the accidental ingestion of *Ascaris lumbricoides* or *Ascaris suum* eggs, which are widespread in the environment. Following ingestion, the larvae emerge in the gastrointestinal tract and utilize the circulatory system to migrate through the liver and into the pulmonary tissues. Within the lungs, the larvae traverse the vascular endothelium to enter the parenchyma for further maturation. They eventually migrate across the alveolar surface, ascend the respiratory tract, and are swallowed to reach their final niche in the small intestine [6–8]. This intricate, two-month migratory phase is a physiological requirement for the development of mature adult worms [9].

In the acute phase, the migration of *Ascaris* larvae through the pulmonary tissues triggers the rapid onset of type-2 inflammation and allergic airway disease. Experimental mouse models demonstrate that the transit of *Ascaris* larvae incites a robust recruitment of type-2 inflammatory cells, characterized by elevated cytokine production, significant airway hyperreactivity, and excessive mucus secretion. While this aggressive immunological recruitment is functionally intended to constrain larval development and limit the total parasite burden, its immediate clinical result is a period of transient, yet severe, allergic inflammation [10].

Additionally, *Ascaris* larvae migration incites enduring pathological changes that extend far beyond the acute phase of infection. In experimental mouse model, *Ascaris* larvae are cleared from the host within 14 days post infection through the intestines [11]. We have previously demonstrated that even a solitary exposure to *Ascaris spp.* can precipitate chronic lung disease in murine models months after infection resolution, manifesting as both persistent type-2 inflammatory pathology and emphysema-like structural remodeling [12]. Notably, this chronic state is characterized by heightened vascular permeability and the development of systemic anemia that persists for months following the resolution of the primary infection [12]. While prior investigations suggested that sequestered helminth antigens might drive these long-term sequelae [13], the precise molecular identity of these signals and the pathways by which they maintain T cell activation have remained elusive.

In the present study, we utilized immunopeptidomics to identify a specific retained peptide derived from the parasite that is continuously presented by MHC class II on pulmonary macrophages to the adaptive immune system. This persistent antigenic signal maintains the activity of epitope-specific T cells, providing a clear molecular link between initial infection and chronic inflammatory pathology. Identification of this retained epitope establishes a potential molecular basis for the chronicity of *Ascaris*-induced disease and highlights a specific target for future therapeutic intervention.

## Results

### Immunopeptidomics identifies a specific *Ascaris* peptide retained in the lung

Our investigation into the specific molecular signatures retained in the lung was informed by the findings of Oliveira et al. [13]. This study demonstrated that *Ascaris* larval migration leaves behind long-lasting parasite antigens sequestered within the lung parenchyma, and specifically within pulmonary macrophage cytoplasm, which provide a continuous source of immune stimulation even after physical parasite clearance. Because pulmonary macrophages are the specialized phagocytes responsible for the uptake and long-term sequestration of such parasite-derived products, we identified them as the most likely cellular reservoir for these persistent peptides.

**Fig 1:**
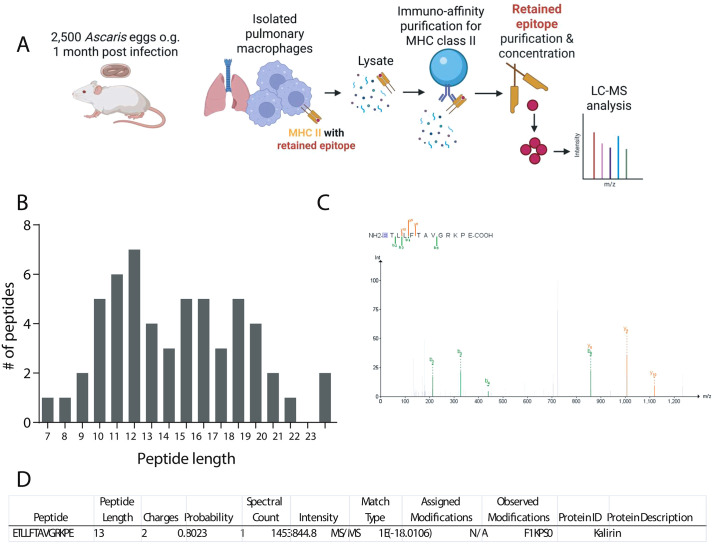
Identification of retained *Ascaris* epitopes in the lung post-infection. **(A)** Schematic of experimental design and immunopeptidomic workflow. **(B)** Length distribution of all 57 peptides identified presented by MHC-II. **(C)** Tandem mass spectrometry fragmentation spectrum of the retained peptide ETLLFTAVGRKPE. **(D)** Peptide identification information provided by Fragpipe analysis. (Fig 1A created in BioRender. Poveda, **C.** (2026) https://BioRender.com/d81rzs7).

Briefly, mice were infected with a standard inoculum of 2,500 *Ascaris suum* eggs via oral gavage. One month after infection, mice were euthanized and lung tissue was harvested. Total pulmonary macrophages were isolated using F4/80 magnetic beads and the cell lysate was prepared (Fig 1A). To identify peptides presented by these isolated macrophages, immunoaffinity purification of the Major Histocompatibility Complex (MHC) class II, which primarily presents peptides derived from extracellular sources, was performed. Purified MHC class II proteins were denatured to release the bound peptides, which were then purified and concentrated. Finally, the collected peptide repertoire was analyzed via Liquid Chromatography-Mass Spectrometry (LC-MS) to identify the retained peptide (Fig 1A).

Immunopeptidomics analysis using proteomes from mouse and *Ascaris* revealed 57 unique peptide presented by MHC class II on pulmonary macrophages (Fig 1A, S1 Table). While the majority of the peptides were derived from the murine host itself, and several peptides existed in proteins from both murine host and *Ascaris,* one single peptide unique to *Ascaris* was identified: **ETLLFTAVGRKPE**, which is clearly evidenced by the MS/MS fragmentation spectrum (Fig 1B, 1C).

Bioinformatic analysis confirmed that this 13-aa peptide is a fragment of the protein Kalirin (Fig 1C, 1D). Kalirin is a Rho guanine nucleotide exchange factor (RhoGEF) that facilitates the transition of small Rho GTPases from an inactive guanosine diphosphate (GDP)-bound state to an active guanosine triphosphate (GTP)-bound state [14]. This transition initiates downstream cascades that regulate the actin cytoskeleton, vesicular trafficking, and neuroendocrine signaling [14, 15]. Notably, this specific sequence is uniquely conserved within *Ascaris spp.*, *Parascaris univalens*, and *Toxocara canis*, but is entirely absent from the host proteome. The discovery of this unique Kalirin-derived peptide represents the first identification of a specific *Ascaris* peptide that remains sequestered within pulmonary macrophages long after the clearance of the primary infection.

### Immunohistochemistry confirms the sequestration of the retained peptide in pulmonary macrophages

**Fig 2:**
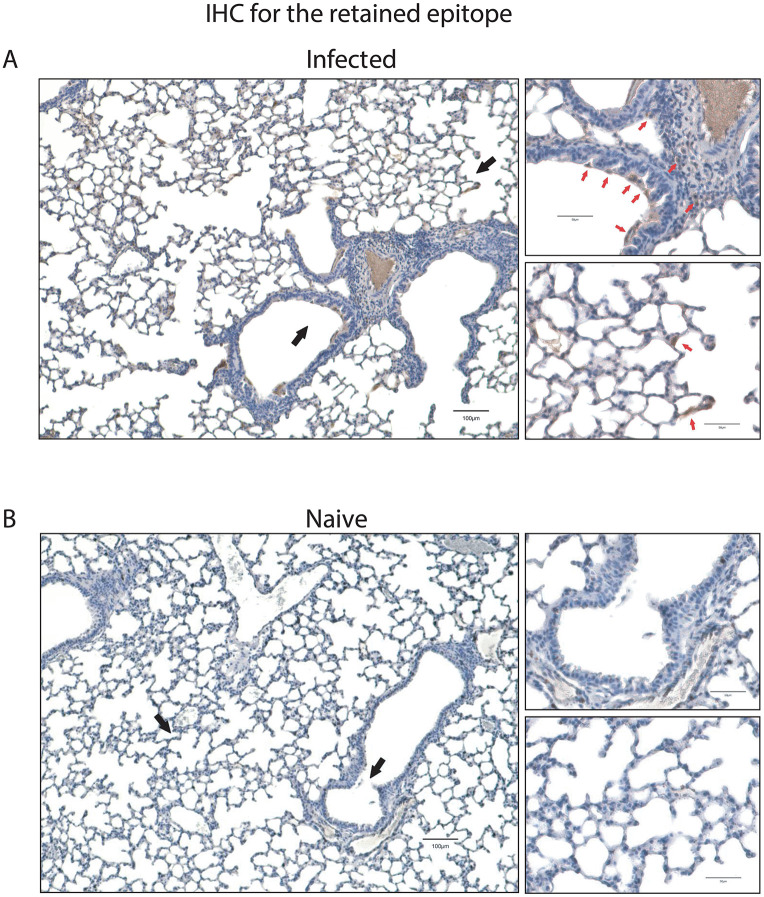
Validation of retained *Ascaris* epitopes in the lung post-infection. Immunohistochemistry was carried out using customized polyclonal antibody against retained epitope on (**A**) lungs from mice 1 month post infection compared to (**B**) naïve control. Black arrows annotate regions selected for higher magnification, while red arrows indicate epitope-positive pulmonary macrophages. (Magnification: 100× and 400 × . Scale bar: 100μm and 50μm. Data are shown as representative of two independent experiments.).

To verify the presence of the Kalirin-derived retained peptide within the lungs post *Ascaris* clearance, we developed a custom polyclonal antibody against the peptide and performed immunohistochemistry (IHC) on lung sections from mice 1 months post infection. We observed notable DAB-positive staining adjacent to the alveolar tissue in the lungs of infected mice (Fig 2A) which was entirely absent in the naïve controls (Fig 2B). Quantification of IHC images revealed that 2.36% of the total lung area was positive for the Kalirin-derived peptide, with the highest density signal localized to the bronchovascular bundle (BVB). Notably, we identified both alveolar and interstitial macrophages containing the retained peptide localized in the alveoli or around the BVB, indicated by the red arrows. This spatial confirmation demonstrates that the retained peptide is specifically sequestered within the pulmonary macrophage niche long after the physical parasite has been eliminated.

### The retained peptide serves as epitope to maintain a specific CD4^+^ T cell population post-clearance

**Fig 3:**
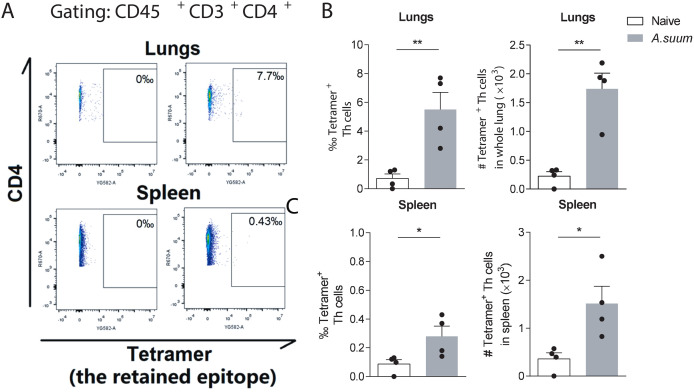
Identification of epitope-specific CD4 + T cells in the lungs. Flow cytometric analysis on live CD45^+^ CD3^+^ CD4^+^ T helper cell that binds to MHC class II tetramer holding the retained epitope. Mean±S.E.M for lungs: 0.7100‰ ± 0.3201, 5.500‰ ± 0.119; for spleen: 0.08713‰ ± 0.02978, 0.2800‰ ± 0.07083) (**A)** Representative flow cytometry gating strategy for MHC class II tetramer. **(B-C)** Permillage and absolute number of tetramer positive T helper cells in (**B**) the lungs and (**C**) the spleen. (n = 4, mean±S.E.M, *p < 0.05, **p < 0.01, using two-tailed Student’s t-test. Data are shown as representative of two independent experiments.).

To determine if the Kalirin-derived retained peptide contributes to chronic T cell activation as an epitope after *Ascaris* clearance, we developed a custom MHC class II tetramer loaded with the peptide. We then performed flow cytometric analysis to identify CD4 + T cells specifically reactive to the retained peptide as epitope in mice one month post-infection. Our analysis revealed a distinct, albeit rare, population of epitope-specific CD4^+^ T cells in both the lungs and the spleen (Fig 3A–3C). These findings demonstrate that the retained epitope serves as a persistent antigenic stimulus that maintains a dedicated T cell repertoire both in the lungs and systemically in the host despite resolution of the initial infection.

## Discussion

Here, we provide the first evidence of a specific retained epitope from *Ascaris* that significantly outlasts the physical presence of the parasite and may contribute to chronic immunological memory. By mapping the pulmonary immunopeptidome, we identified a total of 57 unique peptides (peptides shared within both the mammalian and non-mammalian proteome) and one *Ascaris*-specific peptide, fragment of the RhoGEF protein Kalirin, that are sequestered within the pulmonary macrophage niche following resolution of *Ascaris* infection. The discovery that this single retained epitope maintains a persistent CD4^+^ T cell repertoire suggests that helminth-induced lung disease is not merely a consequence of initial mechanical and chemical tissue damage, but in addition, may manifest chronically by an antigen-driven process that continues long after parasite elimination. This mechanism provides a molecular explanation for the enduring pulmonary morbidity and systemic effects observed in mice even after resolution of infection and may provide an explanation to the high prevalence of non-smoking related chronic lung disease in low- and middle-income countries where *Ascaris* is endemic [16, 17].

In the standard model of pulmonary immunity to extracellular pathogens, antigens are internalized by phagocytes—predominantly pulmonary macrophages—and processed into epitopes for MHC class II presentation. Under normal conditions, this presentation is transient and should conclude shortly after the pathogen is eliminated to prevent chronic inflammation and immune-mediated tissue damage. However, our data show that the *Ascaris*-derived retained epitope persists within the lung chronically post-*Ascaris* larval clearance.

The mechanisms allowing this specific Kalirin-derived retained epitope to bypass typical degradation pathways remain to be fully elucidated. It is possible that the unique structural properties of the Kalirin fragment confer resistance to lysosomal proteases, or that the specialized intracellular environment of pulmonary macrophages—particularly interstitial macrophages—acts as a protective niche [18, 19]. This sequestration could effectively convert a transient innate response into a chronic antigenic stimulus, providing a molecular basis for the persistent immunological remodeling of the lung following the resolution of the larval migration cycle.

As shown in Fig 3, we have identified a distinct, rare population of CD4^+^ T cells reactive to the Kalirin-derived retained epitope. While the specific effector profile of these cells remains under investigation, their persistence months after infection resolution suggests they are central to the maintenance of immune-mediated chronic pulmonary diseases. In typical models of chronic antigen exposure, T cells often transition into an exhausted state characterized by the expression of inhibitory receptors like PD-1 or LAG-3 [20]. However, the progressive nature of the emphysema-like remodeling and vascular permeability observed in our model suggests these epitope-specific T cells may actually contribute to the chronically active and pathogenic environment.

T helper cells are central coordinators of the host immune response to *Ascaris* larval migration. During the acute phase, the transit of larvae through the lung parenchyma incites a robust Type 2 (Th2) response characterized by the recruitment of eosinophils and the production of IL-4, IL-5, and IL-13, which collectively drive acute allergic airway disease and chronic lung pathologies [10, 12]. While this response is intended to constrain larval development, our data suggest that the persistent presence of the Kalirin-derived peptide maintains a specific CD4 + T cell repertoire long after the larvae have cleared. In typical helminth models, sustained type-2 signaling can transition from a wound-healing role into a driver of pathological tissue remodeling [12].

Specifically, these persistent T cells could promote the COPD-like phenotype through the sustained secretion of pro-inflammatory cytokines that recruit secondary effector cells and activate matrix-degrading enzymes. Chronic type-2 signaling is known to stimulate macrophages to release matrix metalloproteinases (MMPs) which can degrade the alveolar architecture and lead to the emphysematous changes as we have shown previously in the *Ascaris* murine model [12]. Furthermore, the localized activity of these T cells around the bronchovascular bundle may disrupt endothelial barrier integrity, providing a mechanistic link to the long-term vascular permeability and subsequent anemia observed in murine lungs months after *Ascaris* infection resolution [12]. The continuous stimulation provided by the retained epitope prevents the restoration of pulmonary homeostasis and transforms a transient larval migration into a driver of sustained pulmonary immune activation.

The discovery of a retained *Ascaris*-specific epitope that persists after parasite clearance could have profound implications for global health strategies. Current World Health Organization guidelines rely heavily on Mass Drug Administration (MDA) using anthelmintic drugs like albendazole and mebendazole. However, these medication strategies primarily target the adult worm and lack significant efficacy against re-infection and migrating larvae. The larval transit through the lungs—and the subsequent deposition of the retained epitope—remains an unaddressed window of pathology.

Our findings suggest that even a successfully treated infection can leave a reservoir of sequestered antigens that continue to fuel chronic pulmonary inflammation. Consequently, clinical management of ascariasis must evolve beyond simple parasite elimination from the infected host to include interventions that either prevent *Ascaris* infection or target the retained epitope within the specialized immune compartments. Future efforts should prioritize the development of therapies or vaccines designed to neutralize these retained molecular signals, thereby preventing the transition from a transient infection to life-long chronic respiratory disease.

The identification of a specific, non-mammalian retained epitope derived from Kalirin offers a promising target for the development of the first preventative or therapeutic vaccine against *Ascaris*-induced pulmonary disease. In addition to previous vaccine efforts that have targeted preventing infection or reducing worm burden, an alternative strategy of vaccine development should evaluate if vaccination against the specific retained epitope could neutralize the “molecular legacy” of larval migration through the lungs. By priming the immune system to recognize and rapidly clear this peptide, such a vaccine could prevent its sequestration within the specialized macrophage niche and prevent long-term pulmonary morbidity. Unlike established vaccine candidates such as As14 or As16 [21], which target larval surface proteins to prevent infection or migration, the Kalirin-derived epitope offers a distinct therapeutic strategy. By targeting a sequestered signal responsible for chronic immune activation, such a vaccine could potentially treat individuals who have already been exposed, effectively neutralizing the persistent antigenic stimulus and preventing the transition to chronic respiratory disease.

While we identified a total of 57 unique peptides (peptides shared within both the mammalian and non-mammalian proteome), only one peptide was *Ascaris*-specific and used for further downstream investigations. The detection of a single dominant *Ascaris*-specific peptide from Kalirin is unusual but may be influenced by the specific binding preferences of the BALB/c I-A^d^ MHC-II allele. Furthermore, our strict bioinformatic filtering—intended to minimize false positives—may have excluded other parasite specific peptides and those with significant homology to host proteins. Certainly, other peptides may play a role in long-term immune activiation, however, the identification of this single high-confidence epitope provides a robust starting point for evaluating the ‘molecular legacy’ of larval migration through the lungs.

While we identified a single, dominant retained epitope from the protein Kalirin, it is also possible that other parasite-derived peptides persist that were lost during the peptide isolation and purification steps or exist at levels below the current limit of detection for LC-MS/MS. Future studies utilizing enrichment techniques may reveal additional components of the antigenic footprint left by migrating larvae. Our data localized the peptide within the specialized macrophage niche, but pulmonary macrophages represent a heterogeneous population consisting of distinct subsets such as alveolar and interstitial macrophages with distinct functions [12]. Given that interstitial macrophages occupy a niche closer to the BVB, they may play a more important role in long-term presentation of the epitope. Further investigation using single-cell proteomics or spatial transcriptomics is required to determine if a specific subset is uniquely responsible for the long-term sequestration of the Kalirin fragment. In addition, further studies on other macrophage-rich organs, such as lymph nodes or livers may reveal other long-term epitope reservoirs that serve as a nidus of on-going immune activation.

We successfully identified a persistent CD4^+^ T cell population reactive to the Kalirin epitope. However, it is important to note that these epitope-specific T cells represent rare events, often occurring at frequencies of less than 0.1% of the total pulmonary CD4^+^ T cells. Consequently, the full transcriptomic and epigenetic profile of these cells remains to be elucidated. The low frequency of these Kalirin-reactive cells necessitated precise gating for identification in individual animals (n = 4); however, conducting a robust ‘deep-dive’ into lineage-specific (i.e., GATA3 for Th2 cells) or tissue-resident memory markers [22] would require large-scale pooling of animals for every individual biological replicate, which was outside the scope of this discovery-phase study. Determining the specific phenotype of these cells will be a critical objective for future investigations utilizing specialized enrichment or single-cell approaches.

While our current findings demonstrate the persistence and immunogenicity of the Kalirin-derived epitope, several functional questions remain. Future studies involving the immunization of animals with the isolated peptide will be critical to evaluate its full potential as a vaccine candidate and its ability to modulate the host immune landscape. Furthermore, assessing the humoral response and antibody kinetics in relation to peptide sequestration may provide complementary insights into how the host maintains immune pressure over time. Finally, the discovery of this persistent epitope opens the door to reinfection models designed to investigate whether this ‘molecular legacy’ influences the dynamics of subsequent parasite exposures. Determining if the prolonged presence of the Kalirin fragment primes the lung for enhanced protection or exacerbated pathology will be essential for the development of targeted clinical interventions against chronic helminth-induced disease.

In conclusion, our study identifies a specific Kalirin-derived retained epitope as a potential significant molecular contributor of chronic pulmonary pathology following *Ascaris* infection. Using immunopeptidomics, we demonstrated that this unique parasite signature is sequestered within the pulmonary macrophage compartment providing a persistent antigenic stimulus that maintains a may chronically active a pathogenic T cell repertoire. These findings establish that the long-term respiratory morbidity associated with ascariasis could be the result of an active, antigen-driven process that continues long after the physical elimination of the parasite. The discovery of this specific epitope reveals a prospective novel vaccine target capable of intercepting the persistent inflammatory signals that characterize the long-term legacy of larval migration. Ultimately, targeting the retained epitope offers a novel therapeutic pathway to decouple initial and repetitive larval exposure from life-long chronic respiratory disease and could represent a significant advancement in the clinical management of neglected tropical diseases.

## Methods

### Ethics statement

All experimental protocols were approved by the Institutional Animal Care and Use Committee of Baylor College of Medicine (AN-9173) and followed federal guidelines.

**Mice**. 8-week-old BALB/c female mice (wildtype) were purchased from Jackson Laboratories (cat: 000651). Upon arrival, mice were randomized into experimental groups (n = 4 per group for flow cytometry and histology; n = 10 mice pooled per replicate for immunopeptidomics). Only female mice were used to ensure consistency in immune responses and *Ascaris suum* infectious burden as previously described [12]. All mice were housed in a vivarium under specific-pathogen-free conditions.

***A. suum* experimental murine model:** *A. suum* eggs were obtained from adult female worms from infected pigs in the Weatherhead laboratory at Baylor College of Medicine. Briefly, adult female worms were isolated and dissected to remove the uterus. The uterus was then strained through a filter to release unembryonated eggs. The eggs were washed with PBS three times and subsequently resuspended in sulfuric acid (0.2N) for 60 days to allow for embryonation. For single infections, BALB/c mice were treated with a single inoculum of 2,500 embryonated *A. suum* eggs via oral gavage or PBS as previously described [8, 11, 23]. The dose of 2,500 A. suum eggs is a standardized inoculum used in murine models to successfully recapitulate the severe allergic airway disease and chronic remodeling observed in human and porcine infections [9, 11]. The *A. suum* larvae migratory cycle in a murine model mimics the life cycle in humans, albeit the larvae do not develop into adult worms in the murine model and are excreted in stool prior to development into adults worms, and has been previously described [10, 11]. Following oral gavage of *A. suum* eggs or PBS, mice were euthanized at 1 month post infection, and lungs and spleens were harvested in preparation for experiments described below.

**Single cell collection and preparation from mouse tissues**: Lungs and spleens were harvested post euthanasia. Lungs were cut into small pieces and incubated in digestion buffer (2mg/ml collagenase (#LS004177, Worthington), 0.04mg/ml DNAse (#10104159001, Sigma) 1, 20% FBS in HBSS) for 1 h at 37°C after which they were deaggregated by pressing through a 40 μM nylon mesh and centrifuged at 400 x g for 5 minutes at 4°C. Alternatively, spleens were deaggregated by directly pressed through a 40 μM nylon mesh and centrifuged at 400 x g for 5 minutes at 4°C. Supernatants were discarded, and 1.5 mL of ACK (Thermofisher scientific, Waltham MA) was added and incubated for 3 min at room temperature for erythrocyte lysis. ACK was then neutralized with 7.5 mL of complete RPMI-1640 (Corning, NY), with 10% FBS and 1% Pen Strep, (Gibco, Waltham MA). The resulting single cell suspension was centrifuged and prepared for further analysis [24, 25].

**Pulmonary macrophage isolation**: Total pulmonary macrophages from lung single cell suspension were isolated using anti-F4/80 microbeads (cat: 130-110-443, Miltenyi Biotec) following the manufacturer’s protocol using LS columns (cat: 130-042-401, Miltenyi Biotec) and MidiMACS separator (cat 130-042-301, Miltenyi Biotec). For the discovery of retained epitopes, pulmonary macrophages were pooled from 10 infected mice to optimize peptide recovery and ensure sufficient coverage of the immunopeptidome. While the LC-MS/MS was conducted as a high-sensitivity discovery run, the resulting epitope signature was validated in subsequent independent experiments.

**Peptide-MHC-II complex purifications**: A detailed protocol describing the purification of MHC-bound peptides for identification by mass spectrometry has been previously described by our laboratory [26]. Briefly, murine pulmonary macrophages were lysed in lysis buffer (1% octyl-β-D-glucopyranoside, 0.25% sodium deoxycholate, 1.25x complete protease inhibitor cocktail, 1 mM PMSF (phenylmethylsulfonyl fluoride), 0.2 mM iodoacetamide, 1 mM EDTA (ethylenediaminetetraacetic acid), 150 mM NaCl, and 20 mM Tris-HCl, pH 8.0) while rotating at 4 °C. After 1 hour, the lysate was centrifuged down for 10 min at 2,000 x g, and the supernatant was subsequently spun down again for 30 min at 16,000 x g at 4 °C. Soluble proteins in supernatant were removed from insoluble, pelleted fraction.

To perform the immunoaffinity purification of the MHC-II peptides, peptide–MHC-II complexes in soluble lysate fraction were immunoprecipitated using 2.5 mg anti-mouse MHC-II (clone M5/114, cat. 14-5321-85, Thermofisher) monoclonal antibodies covalently linked to AminoLink Plus coupling resin (cat. 20501, Thermofisher). The lysate was rotated overnight at 4 °C with the resin. The following day, the resin containing peptide – MHC-II complexes was washed sequentially with 150 mM NaCl in 20 mM Tris pH 8.0, 400 mM NaCl in 20 mM Tris, pH 8.0 (high salt), 150 mM NaCl in 20 mM Tris pH 8.0, and finally 20 mM Tris pH 8.0 (no salt). Peptide–MHC-II complexes were finally eluted using 10% acetic acid. The eluent was further acidified by adding trifluoroacetic acid to a final concentration of 0.5% (v/v).

To separate peptides from MHC molecules, a C18 resin cartridge (cat. 60108, Thermofisher) purification was conducted [27]. The C18 resin was activated with 100% acetonitrile containing 0.1% TFA and then pre-equilibrated with water containing 0.1% TFA. After loading the eluent onto the cartridge, peptides and proteins were allowed to bind, and the resin was washed with 100% water containing 0.1% TFA. Peptides were then eluted from the column using 25% acetonitrile containing 0.1% TFA, while larger proteins including MHC molecules remained bound. Purified peptides were dried overnight in a SpeedVac, and stored at −80 °C.

**Nano-LC-MS/MS analysis**: Nano-LC-MS/MS analysis were carried out on dried purified peptide by **Creative proteomics**. *Peptide Desalting and Preparation*: Following immunoaffinity purification and elution, the peptide samples were desalted using ZipTip C18 columns (Millipore). Briefly, columns were activated with 100% acetonitrile (ACN) and equilibrated with 0.1% trifluoroacetic acid (TFA). Samples were loaded in 0.1% TFA, washed, and eluted in 60% ACN. The resulting eluates were lyophilized to near dryness and resuspended in 20μL of 0.1% formic acid (FA) for subsequent mass spectrometry analysis.

*Liquid Chromatography*: Separation Peptide separation was performed on an Ultimate 3000 nano UHPLC system (Thermo Scientific). The system utilized a two-column setup: a PepMap C18 trapping column (100 Å, 100μm × 2 cm, 5μm) followed by a PepMap C18 analytical column (100 Å, 75μm × 50 cm, 2μm). Mobile phases consisted of 0.1% FA in water (Phase A) and 0.1% FA in ACN (Phase B). Peptides were separated at a flow rate of 250 nL/min using a multi-step linear gradient: 2% to 8% B over 5 min, 8% to 20% B over 60 min, and 20% to 40% B over 33 min.

*Mass spectrometry*: Acquisition The UHPLC was coupled to an Orbitrap Q Exactive HF mass spectrometer equipped with a Nanospray Flex Ion Source. The instrument was operated in Data-Dependent Acquisition (DDA) mode using a Top 20 method. Full MS scans were acquired from 300-1650 m/z at a resolution of 60,000 (at 200 m/z) with an automatic gain control (AGC) target of 3e6. For MS/MS fragmentation, the resolution was set to 15,000 with a normalized collision energy (NCE) of 28%. The isolation window was maintained at 1.4 Th, and dynamic exclusion was set to 30 s to prevent redundant precursor sampling. Precursors with unassigned charges, or charge states of 1 or >6, were excluded from fragmentation. Raw data files are available via ProteomeXchange via the PRIDE partner repository with identifier PXD079009.

**LC-MS/MS data analysis:** The raw mass spectrometry data were analyzed using FragPipe (v23.0) and MSFragger (v4.2) [28]. To identify MHC class II-restricted peptides, MS/MS spectra were searched against a combined database containing both host and parasite proteomes. These included the *Mus musculus* proteome (UniProtKB; accessed 2025/10/16) and the *Ascaris suum* proteome (UniProtKB; accessed 2025/10/16). Using these proteomes a single FASTA file was generated and decoys were added by Fragpipe prior to analysis. Furthermore, all Fragpipe parameters were set at default, including the FDR (false discovery rate) at 1%.

**Polyclonal antibody generation:** A custom polyclonal antibody targeting the retained epitope (ETLLFTAVGRKPE) was generated by GenScript (Piscataway, NJ, USA). Briefly, rabbits were immunized with the synthetic peptide and the resulting antiserum was affinity-purified against the target antigen (ETLLFTAVGRKPE) to ensure high specificity. Polyclonal antibody avidity and specificity were verified by indirect ELISA against the immunizing peptide. According to manufacturer, the polyclonal antibody demonstrated an OD450 from 3.134 to 1.678 at 1:1000–1:512,000 while IgG control showed background at 0.050.

**Histopathology:** Lung tissue from mice 1 month post infection was fixed in 10% neutral-buffered formalin solution, processed and embedded in paraffin. 5 μm sections were cut and immunohistochemistry was carried out. Briefly, slides were deparaffinized, then permeabilized using 0.2% Triton X 100 (X100, Sigma-Aldrich, St. Louis, MO). After that, antigen recovery was completed using Diva Decloaker (DV2004, Biocare Medical, Pacheco, CA), and peroxidase blocked using 3% hydrogen peroxide. Slides were then blocked using 5% bovine serum albumin (A0100-005, Gendepot, Baker, TX) for 1 hour at room temperature, and incubated with primary polyclonal antibody against retained epitope (ETLLFTAVGRKPE, see above) in I-Block solution (T2015, Thermofisher scientific, Waltham MA) overnight at 4 °C. Slides were then incubated and stained using ABC-HRP and DAB kit (PK-6200, SK-4100, Vector Laboratories, Newark, CA). Slides were then counterstained with hematoxylin, dehydrated and mounted [23]. Quantification for DAB staining was carried out via ImageJ (Fiji).

**MHC Class II tetramer synthesis:** To identify CD4^+^ T cells specific for the retained epitope, custom ProT2 MHC Class II Tetramers were utilized (ProImmune, Oxford, UK). The tetramers were generated using H-2 IAd molecules loaded with the synthetic retained peptide. These MHC-peptide complexes were multimerized using R-Phycoerythrin (R-PE) conjugated streptavidin to ensure high-avidity binding to antigen-specific T cell receptors.

**Flow cytometry:** Total lung cells and splenocytes isolated from mice 1 month post infection were stained for flow cytometry. Tissue processing and single cell suspension were described above. Briefly, MHC class II tetramers from above were centrifuged at 14000 × g for 5 minutes at 4°C, and stained the cells for 2 hours in the dark at 37 °C following the manufacturer’s protocol. After that, cells were stained with Live/Dead Fixable Violet (L34964, Thermofisher scientific, Waltham MA) and CD45 (103112, Biolegend, San Diego, CA). For T helper cell staining, cells were stained with CD3, CD4 (100222, 100412, Biolegend, San Diego, CA) [24, 25]. Flow cytometry was performed on an LSRII (BD Biosciences) and data were analyzed using FlowJo software (version 10.0.7; Treestar, Ashland, OR). Additional gating strategy is shown in S1 Fig.

**Statistical analysis:** Data are presented as means ± standard errors of the means. Significant differences relative to PBS-challenged naïve mice are expressed by p values of <0.05, as measured two tailed Student’s t-test. Data normality was confirmed using the Shapiro-Wilk test. Experiments were repeated at least twice. All data points in the manuscript identify biological replicates.

## Supporting information

S1 Table Full list of all 57 identified peptides presented by pulmonary macrophages. (XLSX)

S1 Fig Additional flow cytometry gating for pulmonary T cells. (EPS)
