# Supplementary material for: Identification of a persistent Ascaris-derived Kalirin epitope associated with chronic T cell activation in the lung
Source: PLoS Negl Trop Dis. 2026 Jun 9;20(6):e0014434. doi: 10.1371/journal.pntd.0014434 (PMC13268194; doi:10.1371/journal.pntd.0014434)
Supplement: Attachment — Submitted filename: 2026 PLOS NTD response to reviewer 04-09-26_JWedits.docx [file pntd.0014434.s004.docx]

**Methods**

Reviewer #1: - Incomplete reporting of animal numbers: The manuscript does not specify the number of animals included. Although randomization into longitudinal groups is mentioned, only a single time point (1 month post-infection) is presented in the results. Clarification of group sizes and time points is necessary. And the dataset would greatly benefit from including multiple time points.
- Missing ethical approval information: Animal study approval numbers are not provided.
- Choice of female mice: The rationale for using female mice cites a previous/ and in this one rather unrelated publications on sex differences in asthma incidence. This does not justify sex-specific effects in A. suum infection or related to infectious burden?
- Normality of sulfuric acid: The concentration used for egg embryonation is not specified.
- Peptidomics sample clarification: The number of independent biological replicates and any technical replicates for LC‑MS/MS analysis are not stated. It is unclear whether lung cells were pooled or analyzed individually.

*We thank the reviewer for these methodological critiques. We have updated the Materials and Methods and Results sections to provide the following clarifications:*

- *Animal Numbers and Time Points: We have clarified that this study focuses specifically on the established chronic phase at 30 days post-infection. While mice were randomized at the study's onset, n = 4 biological replicates were used per group for individual flow cytometric and histological analyses.*
- *Ethical Approval: The Institutional Animal Care and Use Committee (IACUC) protocol number (AN-9173) has been added to the Methods section.*
- *Sex Choice and Embryonation: Female BALB/c mice were utilized to ensure consistency in infectious burden and immune responses, as established in previous models (Wu et al., PLOS NTD, 2021 2024 & 2025). Additionally, we have specified the sulfuric acid concentration for egg embryonation as 0.2N.*
- *Peptidomics and Validation Strategy: For the immunopeptidomics discovery phase, pulmonary macrophages were pooled from 10 infected mice to ensure parasite-derived MHC-II ligands reached the limit of detection for high-resolution LC-MS/MS .*
- *Biological Reproducibility: Crucially, this single high-yield discovery was validated in two independent biological cohorts using custom polyclonal antibodies (IHC) and custom MHC-II tetramers (Flow Cytometry) performed on individual mice (n = 4 per group). This distinction between the discovery and validation phases has been clarified in the revised text.*

**Results**

Reviewer #1: Results - Major:
- Lack of translational immunogenicity data: The immunogenic and retention potential of the identified peptide is not assessed, particularly in the context of MHCII haplotypes of natural hosts. This significantly limits translational interpretation.
- Data availability: A complete list of all identified peptides per run, including host-derived peptides, should be made available to support reproducibility and community interest in e.g. host proteins, protein modification during retention. If peptides with mixed sequence identity were detected, it becomes even more critical to demonstrate a causal link between the identified epitope and chronic inflammation.
- Longitudinal analysis: Were peptides assessed at multiple time points post-clearance? Longitudinal peptide detection is essential to distinguish true antigen persistence from delayed clearance, stochastic LC‑MS/MS detection, or secondary antigen release through ongoing tissue damage.
- Tetramer+ cell characterization: What proportion of Tet+ cells falls within Th2 vs. non‑Th2 CD4 subsets? Among Th2 cytokine-producing / CD40L+ cells, what fraction is Tet+? More broadly: how are peptide-specific T cells phenotypically characterized? This refers also to TRM/EM/Eff markers: Without tissue-resident memory T‑cell marker analysis, the claim that retained peptides drive chronic lung inflammation is not supported. Correlating peptide persistence with T‑cell activation signatures would significantly strengthen the central claim.

***Author response:*** *We thank the reviewer for the thorough critique. Regarding the identified issues:*

- *Translational Immunogenicity: We agree that evaluating the MHC-II haplotypes of natural hosts (pigs or humans) is the ultimate goal for vaccine development. However, this study utilized the standardized BALB/c (H-2I^A^d) murine model because it is a well-characterized system for helminth-induced lung pathology and allows for the use of high-precision tools like custom tetramers. Defining the existence of persistent antigen sequestration in this controlled model is a necessary first step before expanding into the high-variability MHC landscapes of natural hosts.*
- *Data Availability: We have now included a Supplemental Table 1 containing the full list of all 57 identified peptides. This transparency ensures reproducibility and allows the community to investigate potential modifications or host-protein involvement in antigen retention.*
- *Longitudinal Analysis and Persistence: In this model, Ascaris larvae are physically cleared from the lungs by 14 days post-infection (Weatherhead et al., Infection and Immunity, 2018; Gazzinelli-Guimaraes et al., Int J Parasitol. 2013). Therefore, our detection of the Kalirin-derived epitope at 30 days post-infection—more than two weeks after the resolution of larval migration—is evidence of true antigen persistence rather than delayed clearance. While future studies will evaluate the decay kinetics of this signal, the current data clearly demonstrate that the epitope significantly outlasts the parasite.*
- *Tetramer+ Cell Characterization: We acknowledge that a deeper phenotypic characterization of the epitope-specific T cells—such as evaluating lineage-specific markers (GATA3) or tissue-resident memory markers (CD69, CD103)—would provide additional mechanistic granularity. However, it is critical to note that these Kalirin-reactive T cells represent rare events, often occurring at frequencies of less than 0.1% (permillage) of the total pulmonary CD4+ population . Consequently, conducting robust sub-phenotyping within this specific Tetramer+ fraction would require large-scale pooling of animals for every individual biological replicate, which was logistically and ethically outside the scope of this discovery-phase study.*
- *To address the reviewer’s concern regarding causality, we have tempered our language throughout the manuscript, shifting the focus from the epitope "driving" the disease to its role in the maintenance of a chronically active T cell repertoire that contributes to ongoing pathology.*

Minor:
- Antibody specificity: Specificity of the polyclonal antibodies is insufficiently validated. No data using A. suum larval material are shown.
- Quantitative histology: The strong, widespread staining pattern in the selected representative sections warrants quantification across different lung regions.
- Tissue specificity of antigen retention: The manuscript does not address whether peptide retention is lung‑macrophage specific.
- LC‑MS/MS sensitivity: The authors may consider optimizing MS detection sensitivity to enhance peptide coverage, especially given the presence of Tet+ T cells.
- Macrophage heterogeneity: Given the distinct roles of alveolar vs. interstitial macrophages in antigen processing, the authors should address which population most likely presents the retained peptides.

***Author response:*** *We thank reviewers for these minor comments.*

- *Antibody Specificity: We have clarified in the Methods that the specificity of the custom polyclonal antibody was rigorously verified via indirect ELISA against the immunizing synthetic peptide, showing high avidity. While we did not include whole A. suum larval lysate, the biological specificity is demonstrated by the complete absence of staining in naïve lung tissue and the localized DAB-positive signals in infected lungs that correspond precisely to the macrophage niches identified in our study.*
- *Quantitative Histology: We appreciate this suggestion and have now included quantification of the DAB-positive staining in the revised manuscript.*
- *Tissue Specificity of Antigen Retention: While our study prioritized the lung as the primary site of Ascaris-induced pathology, we identified epitope-specific T cells in the spleen as well as the lungs. This suggests that while the macrophage reservoir may be concentrated in the lung, the resulting immune response is systemic. We have added a comment to the Discussion noting that future studies will evaluate if other macrophage-rich organs, such as the liver or lymph nodes, also serve as long-term peptide reservoirs.*
- *LC-MS/MS Sensitivity: We utilized a high-resolution Orbitrap Q Exactive HF and the FragPipe platform, which are among the most sensitive tools currently available for immunopeptidomics. While further optimization is always possible, our current approach was sufficiently sensitive to identify a unique, high-confidence parasite epitope that was subsequently validated through multiple orthogonal methods.*
- *Macrophage Heterogeneity: Our histological analysis identified the retained epitope in both alveolar and interstitial macrophages. We have expanded the Discussion to address this point.*
- *We have also fixed the figure issues as noted by the reviewers.*

Reviewer #2: The results are clear, complete and easy to understand. The results are in line with proposed research question. Main article images are easy to understand. Struggled to open the supplementary file and could not analyse it properly.

***Author response:*** *Thank you to the reviewer for this positive feedback regarding the clarity of the manuscript. We have re-uploaded the supplementary file*

Reviewer #3: (No Response)

**Conclusions**

Reviewer #1: Based on the currently presented dataset, the main conclusions of the manuscript are only supported to a limited extent. Several key experimental components required to substantiate the proposed mechanism: such as a comprehensive characterization of peptide‑specific T cells, validation of antigen persistence across multiple tissues/cell niches/cells and time points —are missing or insufficiently resolved. As a result, direct evidence for a causal link between long‑term peptide retention and sustained T‑cell activation during chronic inflammation remains preliminary, and the central claims cannot yet be fully justified by the available results.

The current title implies a causal relationship between a single retained epitope and chronic T‑cell activation. This relationship is currently not experimentally demonstrated. The title should be revised to avoid overstating causality.
Discussion
- Several claims in the discussion extend beyond what the data currently support and should be tempered (overinterpretation). As one example: The supposed “specialized macrophage niche” is not demonstrated; only one APC population was analyzed, without distinguishing macrophage subsets or comparing different tissues.
- Infection dose and translational relevance: The unusually high infection dose should be discussed in the context of natural infections in pigs. It is unclear whether similar prolonged allergic responses occur in the natural host.
- Unexpected detection of a single peptide: The detection of only one retained peptide is surprising. Potential explanations related to mouse genetics, antigen processing pathways, and not only MS sensitivity should be addressed.

***Author response:***

*Causality vs. Contribution: We agree that while our data demonstrate a clear association between the persistent Kalirin-derived epitope and a stable CD4+ T cell population, direct causality requires further functional studies, such as peptide-specific deletion or neutralization. We have revised the title and tempered the language throughout the Abstract, Results, and Discussion to describe the epitope as a potential "significant contributor" to, rather than the "primary driver" of, chronic immune activation*

*The "Specialized Macrophage Niche": Pulmonary macrophages were solely investigated in this discovery manuscript based on previous literature identifying pulmonary macrophages as an immune cell population in the lungs that remains activated longitudinally in the Ascaris murine model post-infection and that have demonstrated evidence of retained Ascaris peptides longitudinally (Wu. PLOS NTD. 2021; Oliveira. Microb Pathog. 2024). Future studies will evaluate the presence of retained epitope in other antigen presenting cells in the lungs, macrophages in other host compartments as well as characterize the selected pulmonary macrophages subsets and their longevity.*

*Infection Dose and Translation: The dose of 2,500 A. suum eggs is a standardized inoculum used in murine models to successfully recapitulate the severe allergic airway disease and chronic remodeling observed in human and porcine infections. Future studies will look at the impact of trickle down doses, retained epitopes in the lung macrophages and the observance of chronic disease to determine if the burden of Ascaris larvae on initial infection dictates epitope retention and chronic lung disease. Additional investigations looking at repeat infection are also planned knowing that lung disease is more pronounced on subsequent infection due to fully activated eosinophils (Oliveira Amorim. PLOS NTD. 2024; Nogueira. PLOS NTD. 2016; Gazzinelli-Guimaraes. JClin Invest. 2019)*

*Unexpected Detection of a Single Peptide: The identification of only one parasite-derived peptide is likely a result of our stringent 1% FDR filtering and the specific binding motifs of the BALB/c I-A^d^ MHC-II molecule. Furthermore, many parasite peptides may have been excluded during bioinformatic processing if they shared high homology with the host (mouse) proteome. We have expanded the Discussion to address these technical and biological factors*

Reviewer #2: Conclusions are well presented and supported by the data. Authors clearly discuss how these findings could be useful in the context of Ascaris control and, therefore, its relevance in Public Health. The limitations of the study are well presented and adequate given the context.
A couple of points that could be of interest should the authors consider them as so:
- Could this retainment of the peptide be due to the model itself because of the inability of development of Ascaris adults? As clearance in this species is mainly in the lungs as well while we have some in the human intestine, could we expect a difference in the level of peptide in the lungs between mouse model and humans?
- It is suggested that this peptide could maybe be an interesting target in vaccination development. Maybe it would be interesting to do a very brief comparison between this and other targets, regarding maybe location or parasite development stage?

***Author response:***

Mouse model vs natural host?: *We agree with the reviewer that it is possible this protein retention is species specific. However, it is confirmed that the timeline for Ascaris larval migration through the host lungs into the trachea in mice correlates with the timeline for Ascaris larval migration in natural hosts such as pigs and humans. While the larvae do not develop into adult worms in the intestines of mice, they do migrate out of the lungs and up the trachea similar to natural hosts (the lungs are not the main region of clearance in mice after a single infection, larvae are eliminated through the intestinal track) suggesting a similar process of epitope retention in the lungs may be happening in pigs and humans as well, a direction we will investigate in the future.*

*On Vaccine Target Comparison: We have expanded the Discussion to briefly compare our Kalirin-derived epitope with existing vaccine candidates. Traditional targets, such as As14 or As16, are primarily surface-expressed larval proteins designed to prevent initial infection or inhibit migration. In contrast, the Kalirin epitope represents a sequestered, non-surface-exposed signal that drives chronic pathology. This distinction positions our epitope as a unique candidate for a therapeutic or "anti-pathology" vaccine specifically designed to neutralize the "molecular legacy" of infection and decouple initial exposure from lifelong respiratory disease*

Reviewer #3: (No Response)

**Editorial and Data Presentation Modifications:**

Use this section for editorial suggestions as well as relatively minor modifications of existing data that would enhance clarity. If the only modifications needed are minor and/or editorial, you may wish to recommend “Minor Revision” or “Accept”.

Reviewer #1: For peptide identification studies, I generally suggest that the raw MS data should be available in publicly accessible repositories.

***Author response:*** *We agree with the reviewer and will make the raw MS data available in publicly accessible repositories.*

Reviewer #2: Address the appearance of DOI in references section (Wu 2024).
Both the full and short title specifically mention epitope while in the end of introduction the authors mention peptide, followed by epitope, and then once again and interchange between peptide and epitope in the results section. Could this be evaluated to make it more coherent? Maybe peptide in the title could make it more appealing/understandable as well for the overall audience?

***Author response:*** *We appreciate the reviewer’s keen observation regarding the interchangeable use of "peptide" and "epitope." In our revision, we utilized the term “peptide” during the discovery and validation phase before validation on specific T cells, and used the term “epitope” after validating responsive T cells.*

Reviewer #3: (No Response)

**Summary and General Comments**

Reviewer #1: The manuscript addresses a conceptually very interesting question. The identification of specific epitopes is of particular importance and may provide novel insights into the mechanisms underlying chronic T‑cell activation following helminth infection.
The overall approach of the manuscript is methodologically sound, and the focuses on the identification as a first step in antigen retention. Tissue antigen depots, delayed antigen degradation, and potential cross-reactivity are only discussed, but might be a point for later studies.
However, to me, the dataset appears preliminary, and the authors themselves acknowledge that several important aspects require further investigation. Based on the current level of evidence, it may be premature to support publication as a full research article; the work might be more suitable as a brief report at this stage or requires substantial major revisions.

***Author response:***  *We appreciate the reviewers comments on the novelty of these finds and potential significance as well as the robust review and suggestions presented by the reviewer to strengthen the manuscript. As this is a discovery manuscript with outcomes that significantly impact our field, we feel that the importance of sharing these findings with our scientific communities is of high importance. Although we plan many future experiments to further characterize the natural history of this epitope and the host immune response longitudinally (following different timepoints, different infectious egg concentrations and in different models), we feel like the findings of this study stand on their own merit and additional studies would be outside the scope of this initial discovery paper.*

Reviewer #2: The study presented aims to address the question of which Ascaris peptides are retained in the lungs and further reinduce inflammatory response. This study follows-up findings from Oliveira et al. 2024 that highlighted this situation but did not identify which specific peptides were responsible. Using mouse models the authors were able to identify one peptide that could be responsible for this. The implications on this are of great significance as this could be an important target for vaccine development that could not only act directly on larvae development but also reduce inflammatory response with further re-infections.
The work is well written and clearly presenting both achievements and limitations. While there were just a few points that could improve, the work stands on its own merit as it is.

***Author response:*** *We appreciate the reviewers comments and support of the importance of our findings in gaining greater insight into Ascaris immunopathology.*

Reviewer #3: The present study identified an Ascaris-specific peptide presented by MHC II on pulmonary macrophages even after larval migration through the lungs and the resolution of infection. The identification of this antigen at a persistent stage, one month after infection, represents a novel finding of considerable scientific relevance, particularly as it contributes to the understanding of the immunological mechanisms involved in disease chronicity and may provide insights into potential therapeutic targets. Although the manuscript is well written and concise, and the methodology is consistent with the proposed objectives, I would like to suggest some points for consideration:

General comments
• Some statements throughout the manuscript appear to overemphasize the findings. In particular, in the Author Summary and in the discussion, certain passages suggest that this mechanism is responsible for persistent inflammation, implying a direct cause-and-effect relationship. However, it is well established that immune responses to helminths are multifactorial, and antigen persistence may be associated not only with sustained immune activation but also with immune modulation or regulatory processes. Therefore, the prolonged presence of this antigen should be interpreted as a potential contributing factor rather than the direct causal mechanism of persistent inflammation.

***Author response:*** *We thank the reviewer for this insightful comment regarding the complex, multifactorial nature of helminth-induced immune responses. We agree that while the identification of a persistent parasite-derived epitope is a novel finding, it should be interpreted as a potential contributing factor to chronic inflammation rather than the sole causal mechanism. We have revised the Author Summary, Introduction, and Discussion to temper our conclusions. Specifically, we have replaced 'drives' and 'definitive mechanism' with 'contributes to' and 'proposed molecular mechanism' throughout the manuscript to better reflect the potential for this persistent antigen to influence—rather than exclusively dictate—the long-term immunological landscape of the lung*

Methodology
• I suggest increasing the experimental sample size, considering that animal model studies typically present biological variability. A sample size of four animals may limit the robustness of the analyses and restrict stronger interpretations of the results.

***Author response:*** *We appreciate the reviewer’s concern regarding biological variability and the robustness of our interpretations. While we utilized a sample size of n = 4 individual mice per group for our flow cytometric and histological analyses, it is important to note that these experiments were repeated at least twice in independent biological cohorts to ensure the reproducibility of our findings. Despite the relatively small sample size, we observed statistically significant differences (p < 0.05 or p < 0.01) in epitope-specific T cell populations compared to naïve controls, suggesting a robust biological effect size. Furthermore, our initial immunopeptidomics discovery utilized a pooled sample of 10 mice to capture a broad representation of the pulmonary immunopeptidome. In accordance with the ethical principle of the 3Rs (Reduction), we believe this sample size is sufficient to support the primary discovery of this novel persistent peptide. We have added a statement to the Discussion acknowledging that future studies focusing on subtle phenotypic shifts or inter-individual variability may benefit from larger experimental cohorts*

• In the section Nano-LC-MS/MS Analysis, the word “Briefly” at the beginning of the sentence appears redundant and could be removed.

***Author response:*** *We have removed the redundant word.*

• In the flow cytometry section, it would be helpful to indicate that the description of tissue processing for this technique was already presented in a previous section (Single cell collection and preparation from mouse tissues), which may facilitate understanding.

***Author response:*** *We have included the indication.*

Results and discussion

• Based on the methodology employed, the authors were able to identify the antigen, detect its presence in the lung parenchyma, and analyze the increase in lymphocyte populations over the investigated period. However, based on the markers used in the flow cytometry analysis, it is only possible to conclude that there is an increase in CD4⁺ T lymphocytes. No lineage-specific markers were included to characterize T helper cell subsets, such as GATA3 for Th2 cells. Furthermore, it is not possible to determine whether this expanded population is functionally activated. To support such an interpretation, activation markers such as CD25, CD69, or CD44 would be necessary.

***Author response:*** *We agree with the reviewer that characterizing the specific T helper cell lineage (e.g., GATA3 for Th2) and activation status (e.g., CD25, CD69, CD44) would provide additional mechanistic granularity. However, it is critical to note that these Kalirin-reactive T cells represent rare events, consistently occurring at frequencies of less than 0.1% (permillage) of the total pulmonary CD4+ population.*

*Consequently, conducting robust sub-phenotyping within this specific tetramer-positive fraction—particularly for intracellular transcription factors like GATA3—would require large-scale animal pooling for every individual biological replicate to achieve sufficient event counts for statistical interpretation. Such an undertaking was logistically and ethically outside the scope of this discovery-phase study.*

*The primary objective and novel finding of this work is the identification of a stable, epitope-specific CD4+ T cell population that persists in the lung 30 days post-infection, long after the physical parasite has been cleared. The statistically significant expansion of these rare cells in individual mice (n = 4) compared to naïve controls (p < 0.01) provides definitive evidence of a persistent antigenic stimulus . We have updated the Discussion to acknowledge that while these cells clearly persist, a comprehensive phenotypic "deep-dive" using specialized enrichment or single-cell approaches is a critical future direction*

• With the data currently presented, it is still difficult to determine how the presence of this antigen is effectively influencing the host immune response. To strengthen these interpretations, additional experiments could be considered alongside the current approach. For instance, immunization of animals with the isolated antigen followed by immunological analyses could help evaluate its immunogenic potential. In addition, assessing the humoral response throughout the course of infection could contribute to a better understanding of the immune dynamics associated with antigen persistence. In this context, measuring antibody levels at different time points during infection and after its resolution could provide complementary information on the maintenance of immune responses over time. Another possibility would be to perform a reinfection model to investigate whether the prolonged presence of this antigen influences the dynamics of subsequent infections.

***Author response:*** *We thank the reviewer for these excellent suggestions to further define the functional impact of the Kalirin-derived epitope. We agree that immunization studies, longitudinal humoral monitoring, and reinfection models are the logical next steps for this research program. However, we believe these experiments represent a distinct and substantial body of work that extends beyond the scope of this initial discovery paper:*

- *Immunogenic Potential: We argue that the immunogenic potential of the Kalirin peptide is already demonstrated by our current data. The fact that we detected a statistically significant expansion of endogenous epitope-specific T cells using custom tetramers in the context of a natural infection—without the use of exogenous adjuvants—provides definitive evidence that this peptide is processed and recognized by the host immune system*
- *Humoral Response: While monitoring antibody levels throughout the course of infection is a compelling direction, the primary focus of this study was the cellular reservoir and the MHC-II immunopeptidome . The identification of a persistent peptide-MHC II complex on macrophages provides a novel cellular mechanism for chronicity that stands independent of the humoral response.*
- *Reinfection Model: We completely agree that the impact of this "antigenic legacy" on subsequent challenges is a critical question. In fact, our laboratory is currently investigating how persistent signals influence host gastric and pulmonary reprogramming during repeat challenges (Wu et al., Plos NTD 2024, 2025). However, performing a full reinfection model with the necessary longitudinal controls would represent a separate study. By providing the first molecular identification of a retained Ascaris epitope, this manuscript establishes the foundational framework necessary for the very immunization and reinfection studies the reviewer suggests. We have updated the Discussion to highlight these as high-priority areas for future investigation.*

• Figure 3A: Is the observed profile homogeneous among the four animals analyzed? It would be helpful to include the mean and standard deviation of the gating percentages, which could facilitate interpretation of the data.

***Author response:*** *We appreciate the reviewer's request for clarification on the homogeneity of the flow cytometric data. As noted, Figure 3A provides a representative gating strategy; the exact quantification and biological variability for all four animals are presented in Figures 3B and 3C. In these panels, we have overlaid individual data points (dots) for each mouse on the bar graphs to allow for direct visualization of the distribution across the cohort. The tight clustering of these points demonstrates the high degree of homogeneity in the epitope-specific T cell populations among the infected animals. For additional clarity, we have updated the legend for Figure 3A to include the mean frequency and SEM of the Tetramer+ gate for the infected group, as requested.*

• Figure 3B: In the legend, A. suum should be written in italics.
***Author response:*** A.suum *is written in italics in Figure 3B*

• In the Discussion, in the sentence beginning with “In typical models of chronic antigen exposure…”, a reference should be included at the end of the statement.

***Author response:*** We have included the reference for this statement (Wherry et al., Nature Reviews Immunology, 2015)

• Additionally, the discussion could be strengthened by including a brief overview of the role of T helper cells in Ascaris infection, particularly considering that this cell population was evaluated in the flow cytometry analyses.

***Author response:*** *We thank the reviewer for the suggestion to provide a broader context for the role of T helper cells in Ascaris infection. We have added a brief overview to the Discussion detailing the transition of the CD4+ T cell response from the acute to the chronic phase. Specifically, we highlight how the hallmark Type 2 (Th2) response—initially essential for larval defense and acute allergic inflammation—likely transitions into a chronic, pathogenic driver of the structural remodeling and emphysema-like disease observed in our model. This context further justifies our focus on the epitope-specific CD4+ T cell population as a key mediator of long-term pulmonary morbidity*

Overall, the study presents an original and relevant finding that contributes to the understanding of the immune response associated with Ascaris infection. The suggestions provided aim to strengthen the interpretation of the results and to better contextualize the findings within the existing literature.

***Author response:*** *We appreciate the reviewers thoughtful and detailed comments. We agree the clarification in the text based on the reviewers suggestions and feedback have strengthened the interpretation of the results and contextualized the findings.*
