# Supplementary material for: Identification of a persistent Ascaris-derived Kalirin epitope associated with chronic T cell activation in the lung
Source: PLoS Negl Trop Dis. 2026 Jun 9;20(6):e0014434. doi: 10.1371/journal.pntd.0014434 (PMC13268194; doi:10.1371/journal.pntd.0014434)
Supplement: Attachment — Submitted filename: 2026 PLOS NTD response to reviewer 05-29-26.docx [file pntd.0014434.s005.docx]

**Editorial and Data Presentation Modifications?**

Reviewer #2: The sentence i the begining of the methods "The dose of 2,500 A. suum eggs (...)" is in italic and should be changed.
Small typo in the begining of the discussion with "may contributes". Should be changed to "may contribute".
Mention where raw MS data will be available.

Reviewer #3: The authors have carefully addressed the points raised in the previous review, responded thoughtfully to the comments, and made modifications to the manuscript in accordance with the suggestions provided. At this stage, I only have minor observations related to spelling and font/style consistency:

Discussion section

At the beginning of paragraph 10, the word while appears in a different font from the rest of the text.
In the final sentence of paragraph 11, there are two periods and this should be corrected.

Methods section

In the A. suum experimental murine model subsection, the sentence “The dose of 2,500 A. suum eggs is a standardized inoculum used in murine models to successfully recapitulate the severe allergic airway disease and chronic remodeling observed in human and porcine infections” appears in a different font from the rest of the text.
In the Peptide-MHC-II complex purifications subsection, please correct the spelling/formatting of 1.25x cOmplete.

*We thank the reviewer for these minor comments. We have revised the manuscript and included the availability of the raw MS data.*
