# Supplementary material for: Identification of a persistent Ascaris-derived Kalirin epitope associated with chronic T cell activation in the lung
Source: PLoS Negl Trop Dis. 2026 Jun 9;20(6):e0014434. doi: 10.1371/journal.pntd.0014434 (PMC13268194; doi:10.1371/journal.pntd.0014434)
Supplement: Peer review history

# Peer review: Decision Letter 0

**DOI:** 10.1371/journal.pntd.0014434.r001

**Authors:** Bruce Rosa

3 Apr 2026

PNTD-D-26-00331

Long-term retention of a single Ascaris epitope promotes chronic T cell activation post-clearance

PLOS Neglected Tropical Diseases

Dear Dr. Weatherhead,

Thank you for submitting your manuscript to PLOS Neglected Tropical Diseases. After careful consideration, we feel that it has merit but does not fully meet PLOS Neglected Tropical Diseases's publication criteria as it currently stands. Therefore, we invite you to submit a revised version of the manuscript that addresses the points raised during the review process.

Please submit your revised manuscript within by Jun 02 2026 11:59PM. If you will need more time than this to complete your revisions, please reply to this message or contact the journal office at plosntds@plos.org. When you're ready to submit your revision, log on to https://www.editorialmanager.com/pntd/ and select the 'Submissions Needing Revision' folder to locate your manuscript file.

Please include the following items when submitting your revised manuscript:

* A letter that responds to each point raised by the editor and reviewer(s). You should upload this letter as a separate file labeled 'Response to Reviewers'. This file does not need to include responses to any formatting updates and technical items listed in the 'Journal Requirements' section below.

* A marked-up copy of your manuscript that highlights changes made to the original version. You should upload this as a separate file labeled 'Revised Manuscript with Track Changes'.

* An unmarked version of your revised paper without tracked changes. You should upload this as a separate file labeled 'Manuscript'.

If you would like to make changes to your financial disclosure, competing interests statement, or data availability statement, please make these updates within the submission form at the time of resubmission. Guidelines for resubmitting your figure files are available below the reviewer comments at the end of this letter.

We look forward to receiving your revised manuscript.

Kind regards,

Bruce A. Rosa

Academic Editor

PLOS Neglected Tropical Diseases

Krystyna Cwiklinski

Section Editor

PLOS Neglected Tropical Diseases

Shaden Kamhawi

co-Editor-in-Chief

PLOS Neglected Tropical Diseases

orcid.org/0000-0003-4304-636XX

Paul Brindley

co-Editor-in-Chief

PLOS Neglected Tropical Diseases

orcid.org/0000-0003-1765-0002

**Additional Editor Comments:**

Please carefully consider the reviewer feedback and revise the manuscript accordingly. In accordance with PLOS NTD guidelines, please provide any relevant animal study approval numbers in addition to the approving institution name already provided. Also, in accordance with PLOS NTD data availability guidelines, please ensure that the raw proteomics data is uploaded and accessible on a public repository, and provide access to processed data in a supplementary table. Additionally, please provide the supplementary figure as a PDF file, with a figure caption, for easier access to readers.

**Journal Requirements:**

1) We do not publish any copyright or trademark symbols that usually accompany proprietary names, eg ©,  ®, or TM  (e.g. next to drug or reagent names). Therefore please remove all instances of trademark/copyright symbols throughout the text, including:

- ® on page: 14.

- TM on page: 12.

2) We have noticed that you have uploaded Supporting Information files, but you have not included a list of legends. Please add a full list of legends for your Supporting Information files after the references list.

3) Figure 1: Please confirm whether you drew the images / clip-art within the figure panels by hand. If you did not draw the images, please provide (a) a link to the source of the images or icons and their license / terms of use; or (b) written permission from the copyright holder to publish the images or icons under our CC BY 4.0 license. Alternatively, you may replace the images with open source alternatives. See these open source resources you may use to replace images / clip-art:

- https://commons.wikimedia.org

- https://openclipart.org/

**Reviewers' Comments:**

Reviewer's Responses to Questions

**Key Review Criteria Required for Acceptance?**

As you describe the new analyses required for acceptance, please consider the following:

**Methods**

-Are the objectives of the study clearly articulated with a clear testable hypothesis stated?

-Is the study design appropriate to address the stated objectives?

-Is the population clearly described and appropriate for the hypothesis being tested?

-Is the sample size sufficient to ensure adequate power to address the hypothesis being tested?

-Were correct statistical analysis used to support conclusions?

-Are there concerns about ethical or regulatory requirements being met?

Reviewer #1: - Incomplete reporting of animal numbers: The manuscript does not specify the number of animals included. Although randomization into longitudinal groups is mentioned, only a single time point (1 month post-infection) is presented in the results. Clarification of group sizes and time points is necessary. And the dataset would greatly benefit from including multiple time points.

- Missing ethical approval information: Animal study approval numbers are not provided.

- Choice of female mice: The rationale for using female mice cites a previous/ and in this one rather unrelated publications on sex differences in asthma incidence. This does not justify sex-specific effects in A. suum infection or related to infectious burden?

- Normality of sulfuric acid: The concentration used for egg embryonation is not specified.

- Peptidomics sample clarification: The number of independent biological replicates and any technical replicates for LC‑MS/MS analysis are not stated. It is unclear whether lung cells were pooled or analyzed individually.

Reviewer #2: The methodology presented in this study is clear even though complex for non-experienced readers. It is both solid and supported by clear background evidence. It highlights the use of mouse model to address the possibility of antigen retention that could lead interesting developments in the control of ascariasis.

The methodology used in this study is clear, adequate and supports the objective of this study.

Reviewer #3: (No Response)

**********

**Results**

-Does the analysis presented match the analysis plan?

-Are the results clearly and completely presented?

-Are the figures (Tables, Images) of sufficient quality for clarity?

Reviewer #1: Results - Major:

- Lack of translational immunogenicity data: The immunogenic and retention potential of the identified peptide is not assessed, particularly in the context of MHCII haplotypes of natural hosts. This significantly limits translational interpretation.

- Data availability: A complete list of all identified peptides per run, including host-derived peptides, should be made available to support reproducibility and community interest in e.g. host proteins, protein modification during retention. If peptides with mixed sequence identity were detected, it becomes even more critical to demonstrate a causal link between the identified epitope and chronic inflammation.

- Longitudinal analysis: Were peptides assessed at multiple time points post-clearance? Longitudinal peptide detection is essential to distinguish true antigen persistence from delayed clearance, stochastic LC‑MS/MS detection, or secondary antigen release through ongoing tissue damage.

- Tetramer+ cell characterization: What proportion of Tet+ cells falls within Th2 vs. non‑Th2 CD4 subsets? Among Th2 cytokine-producing / CD40L+ cells, what fraction is Tet+? More broadly: how are peptide-specific T cells phenotypically characterized? This refers also to TRM/EM/Eff markers: Without tissue-resident memory T‑cell marker analysis, the claim that retained peptides drive chronic lung inflammation is not supported. Correlating peptide persistence with T‑cell activation signatures would significantly strengthen the central claim.

Minor:

- Antibody specificity: Specificity of the polyclonal antibodies is insufficiently validated. No data using A. suum larval material are shown.

- Quantitative histology: The strong, widespread staining pattern in the selected representative sections warrants quantification across different lung regions.

- Tissue specificity of antigen retention: The manuscript does not address whether peptide retention is lung‑macrophage specific.

- LC‑MS/MS sensitivity: The authors may consider optimizing MS detection sensitivity to enhance peptide coverage, especially given the presence of Tet+ T cells.

- Macrophage heterogeneity: Given the distinct roles of alveolar vs. interstitial macrophages in antigen processing, the authors should address which population most likely presents the retained peptides.

-

Figure issues:

Fig.2 IHC image quality: The resolution of immunohistochemistry images should be improved.

Fig. 3A: x-axis label should read Tetramer.

Fig. 3B: Interpretation as “Type 2 cells” is not supported, as no Th2-specific markers were included. Absolute cell counts require clarification (per lung, per lobe, per gram tissue).

Reviewer #2: The results are clear, complete and easy to understand. The results are in line with proposed research question. Main article images are easy to understand. Struggled to open the supplementary file and could not analyse it properly.

Reviewer #3: (No Response)

**********

**Conclusions**

-Are the conclusions supported by the data presented?

-Are the limitations of analysis clearly described?

-Do the authors discuss how these data can be helpful to advance our understanding of the topic under study?

-Is public health relevance addressed?

Reviewer #1: Based on the currently presented dataset, the main conclusions of the manuscript are only supported to a limited extent. Several key experimental components required to substantiate the proposed mechanism: such as a comprehensive characterization of peptide‑specific T cells, validation of antigen persistence across multiple tissues/cell niches/cells and time points —are missing or insufficiently resolved. As a result, direct evidence for a causal link between long‑term peptide retention and sustained T‑cell activation during chronic inflammation remains preliminary, and the central claims cannot yet be fully justified by the available results.

The current title implies a causal relationship between a single retained epitope and chronic T‑cell activation. This relationship is currently not experimentally demonstrated. The title should be revised to avoid overstating causality.

Discussion

- Several claims in the discussion extend beyond what the data currently support and should be tempered (overinterpretation). As one example: The supposed “specialized macrophage niche” is not demonstrated; only one APC population was analyzed, without distinguishing macrophage subsets or comparing different tissues.

- Infection dose and translational relevance: The unusually high infection dose should be discussed in the context of natural infections in pigs. It is unclear whether similar prolonged allergic responses occur in the natural host.

- Unexpected detection of a single peptide: The detection of only one retained peptide is surprising. Potential explanations related to mouse genetics, antigen processing pathways, and not only MS sensitivity should be addressed.

Reviewer #2: Conclusions are well presented and supported by the data. Authors clearly discuss how these findings could be useful in the context of Ascaris control and, therefore, its relevance in Public Health. The limitations of the study are well presented and adequate given the context.

A couple of points that could be of interest should the authors consider them as so:

- Could this retainment of the peptide be due to the model itself because of the inability of development of Ascaris adults? As clearance in this species is mainly in the lungs as well while we have some in the human intestine, could we expect a difference in the level of peptide in the lungs between mouse model and humans?

- It is suggested that this peptide could maybe be an interesting target in vaccination development. Maybe it would be interesting to do a very brief comparison between this and other targets, regarding maybe location or parasite development stage?

Reviewer #3: (No Response)

**********

**Editorial and Data Presentation Modifications?**

Use this section for editorial suggestions as well as relatively minor modifications of existing data that would enhance clarity. If the only modifications needed are minor and/or editorial, you may wish to recommend “Minor Revision” or “Accept”.

Reviewer #1: For peptide identification studies, I generally suggest that the raw MS data should be available in publicly accessible repositories.

Reviewer #2: Address the appearance of DOI in references section (Wu 2024).

Both the full and short title specifically mention epitope while in the end of introduction the authors mention peptide, followed by epitope, and then once again and interchange between peptide and epitope in the results section. Could this be evaluated to make it more coherent? Maybe peptide in the title could make it more appealing/understandable as well for the overall audience?

Reviewer #3: (No Response)

**********

**Summary and General Comments**

Use this section to provide overall comments, discuss strengths/weaknesses of the study, novelty, significance, general execution and scholarship. You may also include additional comments for the author, including concerns about dual publication, research ethics, or publication ethics. If requesting major revision, please articulate the new experiments that are needed.

Reviewer #1: The manuscript addresses a conceptually very interesting question. The identification of specific epitopes is of particular importance and may provide novel insights into the mechanisms underlying chronic T‑cell activation following helminth infection.

The overall approach of the manuscript is methodologically sound, and the focuses on the identification as a first step in antigen retention. Tissue antigen depots, delayed antigen degradation, and potential cross-reactivity are only discussed, but might be a point for later studies.

However, to me, the dataset appears preliminary, and the authors themselves acknowledge that several important aspects require further investigation. Based on the current level of evidence, it may be premature to support publication as a full research article; the work might be more suitable as a brief report at this stage or requires substantial major revisions.

Please find my results related concerns as well as the requested additional experiments/data summarized in the Results section.

Reviewer #2: The study presented aims to address the question of which Ascaris peptides are retained in the lungs and further reinduce inflammatory response. This study follows-up findings from Oliveira et al. 2024 that highlighted this situation but did not identify which specific peptides were responsible. Using mouse models the authors were able to identify one peptide that could be responsible for this. The implications on this are of great significance as this could be an important target for vaccine development that could not only act directly on larvae development but also reduce inflammatory response with further re-infections.

The work is well written and clearly presenting both achievements and limitations. While there were just a few points that could improve, the work stands on its own merit as it is.

Reviewer #3: The present study identified an Ascaris-specific peptide presented by MHC II on pulmonary macrophages even after larval migration through the lungs and the resolution of infection. The identification of this antigen at a persistent stage, one month after infection, represents a novel finding of considerable scientific relevance, particularly as it contributes to the understanding of the immunological mechanisms involved in disease chronicity and may provide insights into potential therapeutic targets.

Although the manuscript is well written and concise, and the methodology is consistent with the proposed objectives, I would like to suggest some points for consideration:

General comments

• Some statements throughout the manuscript appear to overemphasize the findings. In particular, in the Author Summary and in the discussion, certain passages suggest that this mechanism is responsible for persistent inflammation, implying a direct cause-and-effect relationship. However, it is well established that immune responses to helminths are multifactorial, and antigen persistence may be associated not only with sustained immune activation but also with immune modulation or regulatory processes. Therefore, the prolonged presence of this antigen should be interpreted as a potential contributing factor rather than the direct causal mechanism of persistent inflammation.

Methodology

• I suggest increasing the experimental sample size, considering that animal model studies typically present biological variability. A sample size of four animals may limit the robustness of the analyses and restrict stronger interpretations of the results.

• In the section Nano-LC-MS/MS Analysis, the word “Briefly” at the beginning of the sentence appears redundant and could be removed.

• In the flow cytometry section, it would be helpful to indicate that the description of tissue processing for this technique was already presented in a previous section (Single cell collection and preparation from mouse tissues), which may facilitate understanding.

Results and discussion

• Based on the methodology employed, the authors were able to identify the antigen, detect its presence in the lung parenchyma, and analyze the increase in lymphocyte populations over the investigated period. However, based on the markers used in the flow cytometry analysis, it is only possible to conclude that there is an increase in CD4⁺ T lymphocytes. No lineage-specific markers were included to characterize T helper cell subsets, such as GATA3 for Th2 cells. Furthermore, it is not possible to determine whether this expanded population is functionally activated. To support such an interpretation, activation markers such as CD25, CD69, or CD44 would be necessary.

• With the data currently presented, it is still difficult to determine how the presence of this antigen is effectively influencing the host immune response. To strengthen these interpretations, additional experiments could be considered alongside the current approach. For instance, immunization of animals with the isolated antigen followed by immunological analyses could help evaluate its immunogenic potential. In addition, assessing the humoral response throughout the course of infection could contribute to a better understanding of the immune dynamics associated with antigen persistence. In this context, measuring antibody levels at different time points during infection and after its resolution could provide complementary information on the maintenance of immune responses over time. Another possibility would be to perform a reinfection model to investigate whether the prolonged presence of this antigen influences the dynamics of subsequent infections.

• Figure 3A: Is the observed profile homogeneous among the four animals analyzed? It would be helpful to include the mean and standard deviation of the gating percentages, which could facilitate interpretation of the data.

• Figure 3B: In the legend, A. suum should be written in italics.

• In the Discussion, in the sentence beginning with “In typical models of chronic antigen exposure…”, a reference should be included at the end of the statement.

• Additionally, the discussion could be strengthened by including a brief overview of the role of T helper cells in Ascaris infection, particularly considering that this cell population was evaluated in the flow cytometry analyses.

Overall, the study presents an original and relevant finding that contributes to the understanding of the immune response associated with Ascaris infection. The suggestions provided aim to strengthen the interpretation of the results and to better contextualize the findings within the existing literature.

**********

PLOS authors have the option to publish the peer review history of their article (what does this mean?). If published, this will include your full peer review and any attached files.

If you choose “no”, your identity will remain anonymous but your review may still be made public.

**Do you want your identity to be public for this peer review?**  For information about this choice, including consent withdrawal, please see our Privacy Policy.

Reviewer #1: No

Reviewer #2: No

Reviewer #3: No

[NOTE: If reviewer comments were submitted as an attachment file, they will be attached to this email and accessible via the submission site. Please log into your account, locate the manuscript record, and check for the action link "View Attachments". If this link does not appear, there are no attachment files.]

**Figure resubmission:**

While revising your submission, we strongly recommend that you use PLOS’s NAAS tool (https://ngplosjournals.pagemajik.ai/artanalysis) to test your figure files. NAAS can convert your figure files to the TIFF file type and meet basic requirements (such as print size, resolution), or provide you with a report on issues that do not meet our requirements and that NAAS cannot fix.

After uploading your figures to PLOS’s NAAS tool - https://ngplosjournals.pagemajik.ai/artanalysis, NAAS will process the files provided and display the results in the "Uploaded Files" section of the page as the processing is complete. If the uploaded figures meet our requirements (or NAAS is able to fix the files to meet our requirements), the figure will be marked as "fixed" above. If NAAS is unable to fix the files, a red "failed" label will appear above. When NAAS has confirmed that the figure files meet our requirements, please download the file via the download option, and include these NAAS processed figure files when submitting your revised manuscript.

**Reproducibility:**

To enhance the reproducibility of your results, we recommend that authors of applicable studies deposit laboratory protocols in protocols.io, where a protocol can be assigned its own identifier (DOI) such that it can be cited independently in the future. Additionally, PLOS ONE offers an option to publish peer-reviewed clinical study protocols. Read more information on sharing protocols at https://plos.org/protocols?utm_medium=editorial-email&utm_source=authorletters&utm_campaign=protocols

# Author comment: Author response to Decision Letter 1

**DOI:** 10.1371/journal.pntd.0014434.r002

13 Apr 2026

Attachment Submitted filename: 2026 PLOS NTD response to reviewer 04-09-26_JWedits.docx

# Peer review: Decision Letter 1

**DOI:** 10.1371/journal.pntd.0014434.r003

**Authors:** Bruce Rosa

22 May 2026

PNTD-D-26-00331R1

Identification of a persistent Ascaris-derived Kalirin epitope associated with chronic T cell activation in the lung

PLOS Neglected Tropical Diseases

Dear Dr. Weatherhead,

Thank you for submitting your manuscript to PLOS Neglected Tropical Diseases. After careful consideration, we feel that it has merit but does not fully meet PLOS Neglected Tropical Diseases's publication criteria as it currently stands. Therefore, we invite you to submit a revised version of the manuscript that addresses the points raised during the review process.

Please submit your revised manuscript by Jun 21 2026 11:59PM. If you will need more time than this to complete your revisions, please reply to this message or contact the journal office at plosntds@plos.org. When you're ready to submit your revision, log on to https://www.editorialmanager.com/pntd/ and select the 'Submissions Needing Revision' folder to locate your manuscript file.

Please include the following items when submitting your revised manuscript:

* A letter that responds to each point raised by the editor and reviewer(s). You should upload this letter as a separate file labeled 'Response to Reviewers'. This file does not need to include responses to any formatting updates and technical items listed in the 'Journal Requirements' section below.

* A marked-up copy of your manuscript that highlights changes made to the original version. You should upload this as a separate file labeled 'Revised Manuscript with Track Changes'.

* An unmarked version of your revised paper without tracked changes. You should upload this as a separate file labeled 'Manuscript'.

If you would like to make changes to your financial disclosure, competing interests statement, or data availability statement, please make these updates within the submission form at the time of resubmission. Guidelines for resubmitting your figure files are available below the reviewer comments at the end of this letter.

As the corresponding author, your ORCID iD is verified in the submission system and will appear in the published article. PLOS supports the use of ORCID, and we encourage all coauthors to register for an ORCID iD and use it as well. Please encourage your coauthors to verify their ORCID iD within the submission system before final acceptance, as unverified ORCID iDs will not appear in the published article. Only the individual author can complete the verification step; PLOS staff cannot verify ORCID iDs on behalf of authors.

We look forward to receiving your revised manuscript.

Kind regards,

Bruce A. Rosa

Academic Editor

PLOS Neglected Tropical Diseases

Krystyna Cwiklinski

Section Editor

PLOS Neglected Tropical Diseases

Shaden Kamhawi

co-Editor-in-Chief

PLOS Neglected Tropical Diseases

orcid.org/0000-0003-4304-636XX

Paul Brindley

co-Editor-in-Chief

PLOS Neglected Tropical Diseases

orcid.org/0000-0003-1765-0002

**Additional Editor Comments:**

We have received feedback on the revision from two of the reviewers, who have suggested some additional minor revisions to be considered.

Additionally, in accordance with Reviewer 1's suggestion, please ensure that the raw MS proteomics datasets are uploaded to an appropriate repository, and provide accession information in the next resubmission, as this is required by PLOS NTD Data Availability requirements. If you do not want to make the data immediately public, then a reviewer link should be provided so that it can be verified.

**Reviewers' comments:**

Reviewer's Responses to Questions

**Key Review Criteria Required for Acceptance?**

As you describe the new analyses required for acceptance, please consider the following:

**Methods**

-Are the objectives of the study clearly articulated with a clear testable hypothesis stated?

-Is the study design appropriate to address the stated objectives?

-Is the population clearly described and appropriate for the hypothesis being tested?

-Is the sample size sufficient to ensure adequate power to address the hypothesis being tested?

-Were correct statistical analysis used to support conclusions?

-Are there concerns about ethical or regulatory requirements being met?

Reviewer #2: (No Response)

Reviewer #3: (No Response)

**********

**Results**

-Does the analysis presented match the analysis plan?

-Are the results clearly and completely presented?

-Are the figures (Tables, Images) of sufficient quality for clarity?

Reviewer #2: (No Response)

Reviewer #3: (No Response)

**********

**Conclusions**

-Are the conclusions supported by the data presented?

-Are the limitations of analysis clearly described?

-Do the authors discuss how these data can be helpful to advance our understanding of the topic under study?

-Is public health relevance addressed?

Reviewer #2: (No Response)

Reviewer #3: (No Response)

**********

**Editorial and Data Presentation Modifications?**

Use this section for editorial suggestions as well as relatively minor modifications of existing data that would enhance clarity. If the only modifications needed are minor and/or editorial, you may wish to recommend “Minor Revision” or “Accept”.

Reviewer #2: The sentence i the begining of the methods "The dose of 2,500 A. suum eggs (...)" is in italic and should be changed.

Small typo in the begining of the discussion with "may contributes". Should be changed to "may contribute".

Mention where raw MS data will be available.

Reviewer #3: The authors have carefully addressed the points raised in the previous review, responded thoughtfully to the comments, and made modifications to the manuscript in accordance with the suggestions provided. At this stage, I only have minor observations related to spelling and font/style consistency:

Discussion section

At the beginning of paragraph 10, the word while appears in a different font from the rest of the text.

In the final sentence of paragraph 11, there are two periods and this should be corrected.

Methods section

In the A. suum experimental murine model subsection, the sentence “The dose of 2,500 A. suum eggs is a standardized inoculum used in murine models to successfully recapitulate the severe allergic airway disease and chronic remodeling observed in human and porcine infections” appears in a different font from the rest of the text.

In the Peptide-MHC-II complex purifications subsection, please correct the spelling/formatting of 1.25x cOmplete.

**********

**Summary and General Comments**

Use this section to provide overall comments, discuss strengths/weaknesses of the study, novelty, significance, general execution and scholarship. You may also include additional comments for the author, including concerns about dual publication, research ethics, or publication ethics. If requesting major revision, please articulate the new experiments that are needed.

Reviewer #2: The changes made to the manuscript based on the suggestions by the others reviewers improved the overall quality of the paper. I want to commend both the other reviewers for their suggestions and the authors for accurately addressing them.

Reviewer #3: (No Response)

**********

PLOS authors have the option to publish the peer review history of their article (what does this mean?). If published, this will include your full peer review and any attached files.

If you choose “no”, your identity will remain anonymous but your review may still be made public.

**Do you want your identity to be public for this peer review?**  For information about this choice, including consent withdrawal, please see our Privacy Policy.

Reviewer #2: No

Reviewer #3: No

[NOTE: If reviewer comments were submitted as an attachment file, they will be attached to this email and accessible via the submission site. Please log into your account, locate the manuscript record, and check for the action link "View Attachments". If this link does not appear, there are no attachment files.]

**Figure resubmission:**

While revising your submission, we strongly recommend that you use PLOS’s NAAS tool (https://ngplosjournals.pagemajik.ai/artanalysis) to test your figure files. NAAS can convert your figure files to the TIFF file type and meet basic requirements (such as print size, resolution), or provide you with a report on issues that do not meet our requirements and that NAAS cannot fix.

After uploading your figures to PLOS’s NAAS tool - https://ngplosjournals.pagemajik.ai/artanalysis, NAAS will process the files provided and display the results in the "Uploaded Files" section of the page as the processing is complete. If the uploaded figures meet our requirements (or NAAS is able to fix the files to meet our requirements), the figure will be marked as "fixed" above. If NAAS is unable to fix the files, a red "failed" label will appear above. When NAAS has confirmed that the figure files meet our requirements, please download the file via the download option, and include these NAAS processed figure files when submitting your revised manuscript.

**Reproducibility:**

To enhance the reproducibility of your results, we recommend that authors of applicable studies deposit laboratory protocols in protocols.io, where a protocol can be assigned its own identifier (DOI) such that it can be cited independently in the future. Additionally, PLOS ONE offers an option to publish peer-reviewed clinical study protocols. Read more information on sharing protocols at https://plos.org/protocols?utm_medium=editorial-email&utm_source=authorletters&utm_campaign=protocols

# Author comment: Author response to Decision Letter 2

**DOI:** 10.1371/journal.pntd.0014434.r004

1 Jun 2026

Attachment Submitted filename: 2026 PLOS NTD response to reviewer 05-29-26.docx

# Editor report: Decision Letter 2

**DOI:** 10.1371/journal.pntd.0014434.r005

**Authors:** Bruce Rosa

2 Jun 2026

Dear Dr. Weatherhead,

We are pleased to inform you that your manuscript 'Identification of a persistent Ascaris-derived Kalirin epitope associated with chronic T cell activation in the lung' has been provisionally accepted for publication in PLOS Neglected Tropical Diseases.

Before your manuscript can be formally accepted you will need to complete some formatting changes, which you will receive in a follow up email. A member of our team will be in touch with a set of requests.

Please note that your manuscript will not be scheduled for publication until you have made the required changes, so a swift response is appreciated.

IMPORTANT: The editorial review process is now complete. PLOS will only permit corrections to spelling, formatting or significant scientific errors from this point onwards. Requests for major changes, or any which affect the scientific understanding of your work, will cause delays to the publication date of your manuscript.

Should you, your institution's press office or the journal office choose to press release your paper, you will automatically be opted out of early publication. We ask that you notify us now if you or your institution is planning to press release the article. All press must be co-ordinated with PLOS.

Thank you again for supporting Open Access publishing; we are looking forward to publishing your work in PLOS Neglected Tropical Diseases.

Best regards,

Bruce A. Rosa

Academic Editor

PLOS Neglected Tropical Diseases

Krystyna Cwiklinski

Section Editor

PLOS Neglected Tropical Diseases

Shaden Kamhawi

co-Editor-in-Chief

PLOS Neglected Tropical Diseases

orcid.org/0000-0003-4304-636XX

Paul Brindley

co-Editor-in-Chief

PLOS Neglected Tropical Diseases

orcid.org/0000-0003-1765-0002

***********************************************************

The authors have addressed all reviewer concerns

p.p1 {margin: 0.0px 0.0px 0.0px 0.0px; line-height: 16.0px; font: 14.0px Arial; color: #323333; -webkit-text-stroke: #323333}span.s1 {font-kerning: none

# Editor report: Acceptance letter

**DOI:** 10.1371/journal.pntd.0014434.r006

**Authors:** Bruce Rosa

Dear Dr. Weatherhead,

We are delighted to inform you that your manuscript, "Identification of a persistent Ascaris-derived Kalirin epitope associated with chronic T cell activation in the lung," has been formally accepted for publication in PLOS Neglected Tropical Diseases.

We have now passed your article onto the PLOS Production Department who will complete the rest of the publication process. All authors will receive a confirmation email upon publication.

The corresponding author will soon be receiving a typeset proof for review, to ensure errors have not been introduced during production. Please review the PDF proof of your manuscript carefully, as this is the last chance to correct any scientific or type-setting errors. Please note that major changes, or those which affect the scientific understanding of the work, will likely cause delays to the publication date of your manuscript. Note: Proofs for Front Matter articles (Editorial, Viewpoint, Symposium, Review, etc...) are generated on a different schedule and may not be made available as quickly.

Soon after your final files are uploaded, the early version of your manuscript will be published online unless you opted out of this process. The date of the early version will be your article's publication date. The final article will be published to the same URL, and all versions of the paper will be accessible to readers.

For Research Articles, you will receive an invoice from PLOS for your publication fee after your manuscript has reached the completed accept phase. If you receive an email requesting payment before acceptance or for any other service, this may be a phishing scheme. Learn how to identify phishing emails and protect your accounts at https://explore.plos.org/phishing.

Thank you again for supporting open-access publishing; we are looking forward to publishing your work in PLOS Neglected Tropical Diseases.

Best regards,

Shaden Kamhawi

co-Editor-in-Chief

PLOS Neglected Tropical Diseases

Paul Brindley

co-Editor-in-Chief

PLOS Neglected Tropical Diseases
